# Cannabinoid Hyperemesis Syndrome: Public Health Implications and a Novel Model Treatment Guideline

**DOI:** 10.5811/westjem.2017.11.36368

**Published:** 2017-11-08

**Authors:** Jeff Lapoint, Seth Meyer, Charles K. Yu, Kristi L. Koenig, Roneet Lev, Sayone Thihalolipavan, Katherine Staats, Christopher A. Kahn

**Affiliations:** * Kaiser Permanente, San Diego Medical Center, Department of Emergency Medicine, San Diego, California; † County of San Diego, Health & Human Services Agency, Emergency Medical Services, San Diego, California; ‡ County of San Diego, Health & Human Services Agency, Public Health, San Diego, California; § University of California Irvine, Department of Emergency Medicine, Orange, California; ¶ Scripps Mercy Hospital, Emergency Department, San Diego, California; || University of California, San Diego, Department of Emergency Medicine, San Diego, California

## Abstract

**Introduction:**

Cannabinoid hyperemesis syndrome (CHS) is an entity associated with cannabinoid overuse. CHS typically presents with cyclical vomiting, diffuse abdominal pain, and relief with hot showers. Patients often present to the emergency department (ED) repeatedly and undergo extensive evaluations including laboratory examination, advanced imaging, and in some cases unnecessary procedures. They are exposed to an array of pharmacologic interventions including opioids that not only lack evidence, but may also be harmful. This paper presents a novel treatment guideline that highlights the identification and diagnosis of CHS and summarizes treatment strategies aimed at resolution of symptoms, avoidance of unnecessary opioids, and ensuring patient safety.

**Methods:**

The San Diego Emergency Medicine Oversight Commission in collaboration with the County of San Diego Health and Human Services Agency and San Diego Kaiser Permanente Division of Medical Toxicology created an expert consensus panel to establish a guideline to unite the ED community in the treatment of CHS.

**Results:**

Per the consensus guideline, treatment should focus on symptom relief and education on the need for cannabis cessation. Capsaicin is a readily available topical preparation that is reasonable to use as first-line treatment. Antipsychotics including haloperidol and olanzapine have been reported to provide complete symptom relief in limited case studies. Conventional antiemetics including antihistamines, serotonin antagonists, dopamine antagonists and benzodiazepines may have limited effectiveness. Emergency physicians should avoid opioids if the diagnosis of CHS is certain and educate patients that cannabis cessation is the only intervention that will provide complete symptom relief.

**Conclusion:**

An expert consensus treatment guideline is provided to assist with diagnosis and appropriate treatment of CHS. Clinicians and public health officials should identity and treat CHS patients with strategies that decrease exposure to opioids, minimize use of healthcare resources, and maximize patient safety.

## INTRODUCTION

Cannabis is the most widely used illicit substance in the United States. In 2014, 22.2 million Americans 12 years of age and older reported current cannabis use.^1^ The rapidly changing political landscape surrounding cannabis use has the potential to increase these numbers dramatically. Twenty-nine states and the District of Columbia have legalized medicinal use of cannabis.^2^ In addition, as of 2017 California, seven other states and the District of Columbia have legalized recreational use of marijuana.^3^ The incidence of CHS and other marijuana-related emergency department (ED) visits has increased significantly in states where marijuana has been legalized.^4^ A study published in 2016 evaluating the effects of cannabis legalization on EDs in the state of Colorado found that visits for cyclic vomiting—which included CHS in this study—have doubled since legalization.^5^

Despite the syndrome’s increasing prevalence, many physicians are unfamiliar with its diagnosis and treatment. CHS is marked by symptoms that can be refractory to standard antiemetics and analgesics.^6^,^7^ Notwithstanding increasing public health concerns about a national opioid epidemic and emerging guidelines advocating non-opioid alternatives for management of painful conditions, these patients are frequently treated with opioids.^6^,^8^,^9^ In light of the public health implications of a need to reduce opioid use when better alternatives exist, this paper describes the current state of knowledge about CHS and presents a novel model treatment guideline that may be useful to frontline emergency physicians and other medical providers who interface with these patients. The expert consensus process used to develop the model guideline is also described.

## CANNABINOID HYPEREMESIS SYNDROME

CHS is a condition defined by symptoms including significant nausea, vomiting, and abdominal pain in the setting of chronic cannabis use.^7^ Cardinal diagnostic characteristics associated with CHS include regular cannabis use, cyclic nausea and vomiting, and compulsive hot baths or showers with resolution of symptoms after cessation of cannabis use. Cyclical vomiting syndrome (CVS) is a related condition consisting of symptoms of relentless vomiting and retching. While CHS patients present with similar symptoms to those with CVS, associated cannabis use is required to make the diagnosis.

CHS patients present to the ED with non-specific symptoms that are similar to other intra-abdominal conditions. These patients command substantial ED and hospital resources. In a small multicenter ambispective cohort study by Perrotta et al., the mean number of ED visits and hospital admissions for 20 suspected CHS patients identified over a two-year period was 17.3 ± 13.6 and 6.8 ± 9.4 respectively.^10^ These patients frequently undergo expensive and non-diagnostic abdominal imaging studies. In the Perrotta study, the mean number of abdominal computed tomography scans and abdominal/pelvic ultrasounds per patient was 5.3 ± 4.1 and 3.8 ± 3.6 respectively. In addition to a contribution to ED crowding by unnecessary prolonged stays to perform diagnostic testing, patients are exposed to potential side effects of medications, peripheral intravenous lines, and procedures such as endoscopies and abdominal surgeries.^7^,^11^,^12^ While treating physicians often administer opioid analgesics and antiemetics, symptom relief is rarely achieved with this strategy. In fact, there is evidence to suggest opioids may exacerbate symptoms.^6^,^7^

**Population Health Research Capsule**

What do we already know about this issue?

Recurrent patient presentations for abdominal pain with nausea/vomiting associated with chronic cannabinoid use may represent cannabinoid hyperemesis syndrome (CHS).

What was the research question?

Investigators sought to create a consensus guideline for rapid identification and opioid-sparing treatment of patients with CHS.

What was the major finding of the study?

Researchers created a concise expert consensus CHS guideline focusing on avoiding opioid analgesia and unnecessary work-ups.

How does this improve population health?

The CHS treatment guideline will assist frontline clinicians to reduce use of unnecessary healthcare resources and promote safe prescribing to help mitigate contributing to the opioid crisis.

## PATHOPHYSIOLOGY OF CHS

The pathophysiology of CHS is unclear.^7^ Paradoxically, there are long-recognized *antiemetic* effects of cannabis, thus leading to its approved use for treatment of nausea and vomiting associated with chemotherapy and appetite stimulation in HIV/AIDS patients. The factors leading to the development of CHS among only a portion of chronic marijuana users are not well understood. Basic science research has identified two main cannabinoid receptors: CB_1_ and CB_2_, with CB_1_ receptors primarily in the central nervous system, and CB_2_ receptors primarily in peripheral tissues. This categorization has recently been challenged and researchers have identified CB_1_ receptors in the gastrointestinal tract.^7^,^9^ Activity at the CB_1_ receptor is believed to be responsible for many of the clinical effects of cannabis use, including those related to cognition, memory, and nausea/vomiting.^13^ Scientists hypothesize that CHS may be secondary to dysregulation of the endogenous cannabinoid system by desensitization or downregulation of cannabinoid receptors.^14^,^15^ Some investigators have postulated that disruption of peripheral cannabinoid receptors in the enteric nerves may slow gastric motility, precipitating hyperemesis.^16^,^17^

Relief of CHS symptoms with very hot water (greater than 41°C) has highlighted a peripheral tissue receptor called TRPV_1_, a G-protein coupled receptor that has been shown to interact with the endocannabinoid system, but is also the only known capsaicin receptor.^13^,^18^ This has led some to advocate for the use of topical capsaicin cream in the management of acute CHS.^18^–^21^

## PRESENTATION AND DIAGNOSIS

A systematic review of CHS by Sorensen et al.^7^ identified major diagnostic characteristics in patients with CHS as the following:

- History of regular cannabis use for over a year (74.8%)
- At least weekly cannabis use (97.4%)
- Severe nausea and vomiting (100%)
- Abdominal pain (85.1%)
- Vomiting that recurs in a cyclic pattern over months (100%)
- Resolution of symptoms after stopping cannabis (96.8%)
- Compulsive hot baths/showers with symptom relief (92.3%)
- Male predominance (72.9%)

Sorensen et al. identified seven diagnostic frameworks, with significant overlap among characteristics listed by the various authors; however, there was no specific mention of how many of the above features are required for diagnosis. Those with the highest sensitivity include at least weekly cannabis use for greater than one year, severe nausea and vomiting that recurs in cyclic patterns over months usually accompanied by abdominal pain, resolution of symptoms after cannabis cessation, and compulsive hot baths/showers with symptom relief. Clinicians should consider other causes of abdominal pain, nausea and vomiting to avoid misdiagnosis.

Abdominal pain is classically generalized and diffuse in nature. CHS is primarily associated with inhalation of cannabis, though it is independent of formulation and can be seen with incineration of plant matter (traditional smoking), vaporized formulations (e-cigarettes), waxes or oils, and synthetic cannabinoids. At the time of this writing, there have been no reported cases associated with edible marijuana. Episodes generally last 24–48 hours, but may last up to 7–10 days. Patients who endorse relief with very hot water will sometimes report spending hours in the shower.^11^,^12^ Many of these patients will have had multiple presentations to the ED with previously negative workups, including laboratory examinations and advanced imaging.^10^ Clinicians should assess for the presence of CHS in otherwise healthy, young, non-diabetic patients presenting with a previous diagnosis of gastroparesis.

Laboratory test results are frequently non-specific. If patients present after a protracted course of nausea and vomiting, there may be electrolyte derangements, ketonuria, or other signs of dehydration. Mild leukocytosis is common. If patients deny cannabis use but suspicion remains high, a urine drug screen should be considered. Imaging should be avoided, especially in the setting of a benign abdominal examination, as there are no specific radiological findings suggestive of the diagnosis.

## PROCESS TO DEVELOP CHS TREATMENT GUIDELINE

Recognizing the public health concerns surrounding the opioid epidemic and the increasing frequency of ED presentations of CHS, the San Diego Emergency Medicine Oversight Commission in collaboration with the County of San Diego Health and Human Services Agency and San Diego Kaiser Permanente Division of Medical Toxicology established an expert consensus panel to create a guideline to unite the regional ED community in the treatment of CHS. The goal of the guideline is to raise awareness of how to recognize and treat CHS, which will allow providers to avoid opioids, radiation, and invasive procedures for CHS patients. Expert panel members engaged in an iterative process to provide evidence-based input into the draft guideline until complete consensus was achieved. The results of this initiative are presented here (Figure).

## TREATMENT OF CHS

Per the expert consensus guideline, once the diagnosis of CHS has been made and there is a low suspicion for other acute diagnoses, treatment should focus on symptom relief and education on the need for cannabis cessation. Capsaicin is a readily available topical preparation that is reasonable to employ as first line treatment.^18^,^20^ While this recommendation is made based on very limited data including a few small case series, capsaicin is inexpensive, has a low risk side-effect profile, makes mechanistic sense, and is well tolerated.^20^,^22^ Conversely, there are no data demonstrating efficacy of opioids for CHS. Capsaicin 0.075% can be applied to the abdomen or the backs of the arms. If the patients can identify regions of their bodies where hot water provides symptom relief, those areas should be prioritized for capsaicin application. Patients should be advised that capsaicin may be uncomfortable initially, but then should rapidly mimic the relief that they receive with hot showers. Additionally patients must be counseled to avoid application near the face, eyes, genitourinary region, and other areas of sensitive skin, not to apply capsaicin to broken skin, and not to use occlusive dressings over the applied capsaicin. Patients can be discharged home with capsaicin, advising application three or four times a day as needed. If capsaicin is not readily available, but there is a shower available in the ED, patients can be advised to shower with hot water to provide relief. Educate patients to use caution to avoid thermal injury, as there are reports of patients spending as long as four hours at a time in hot showers.^11^

Other possible therapeutic interventions include administration of antipsychotics such as haloperidol 5 mg IV/IM or olanzapine 5 mg IV/IM or ODT, which have been described to provide complete symptom relief in case reports.^23^,^24^ Conventional antiemetics, including antihistamines (diphenhydramine 25–50 mg IV), serotonin antagonists (ondansetron 4–8 mg IV), dopamine antagonists (metoclopramide 10 mg IV), and benzodiazepines can be used, though reports of effectiveness are mixed.^7^,^9^ Provide intravenous fluids and electrolyte replacement as indicated. Avoid opioids if the diagnosis of CHS is certain.

Clinicians should inform patients that their symptoms are directly related to continued use of cannabis. They should further advise patients that immediate cessation of cannabis use is the only method that has been shown to completely resolve symptoms. Reassure patients that symptoms resolve with cessation of cannabinoid use and that full resolution can take anywhere from 7–10 days of abstinence.^7^ Educate patients that symptoms may return with re-exposure to cannabis. Provide clear documentation in the medical record to assist colleagues with confirming a diagnosis, as these patients will frequently re-present to the ED.

## CONCLUSION

The incidence of cannabinoid hyperemesis syndrome in patients presenting to U.S. emergency departments is increasing. Awareness of the syndrome, along with education regarding diagnostic criteria and treatment options, may help avoid increased costs of and potential harms from testing for other conditions while providing more targeted and definitive treatment for CHS patients. Furthermore, lengths of stay are reduced when unnecessary testing is avoided. As highlighted by the public health opioid crisis, emergency physicians have a responsibility to prescribe opioids only for conditions where they would benefit patients.^25^ A novel CHS treatment guideline is presented to assist frontline clinicians with managing this increasingly common condition.

## Figures and Tables

**Figure:**
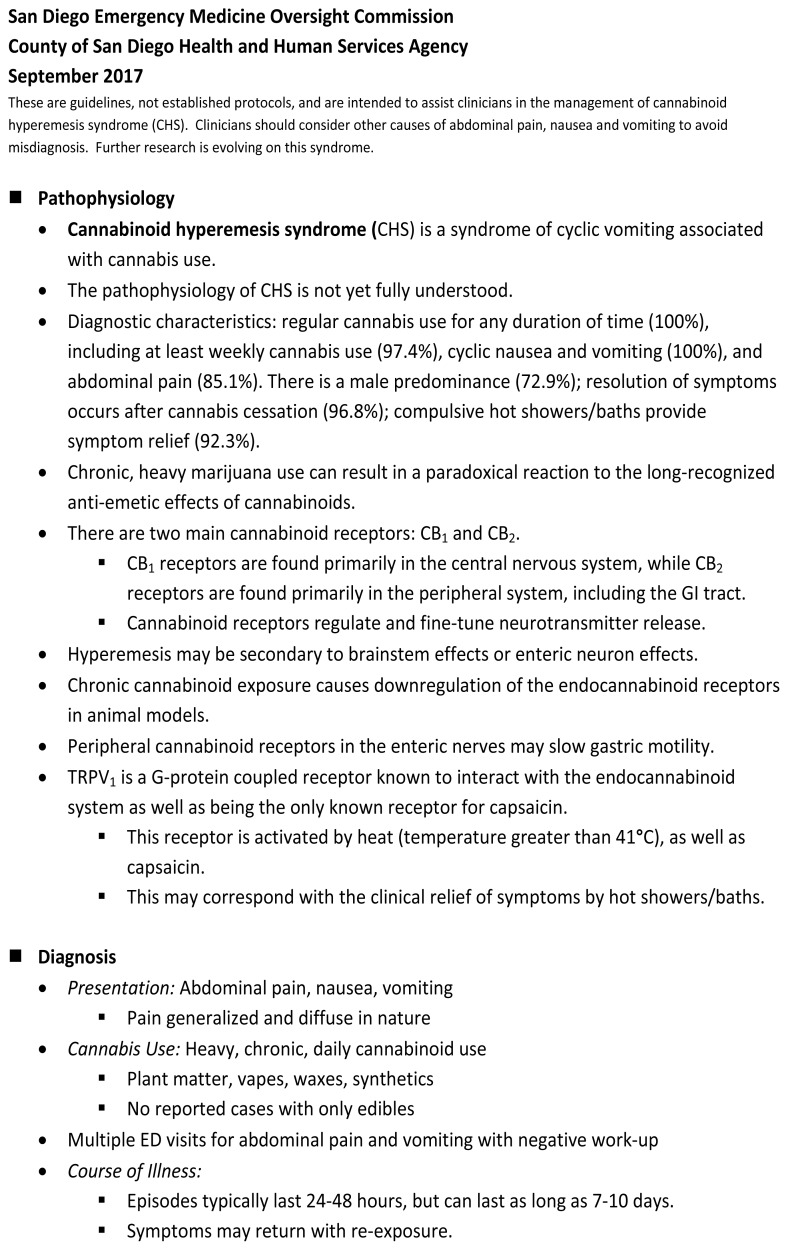

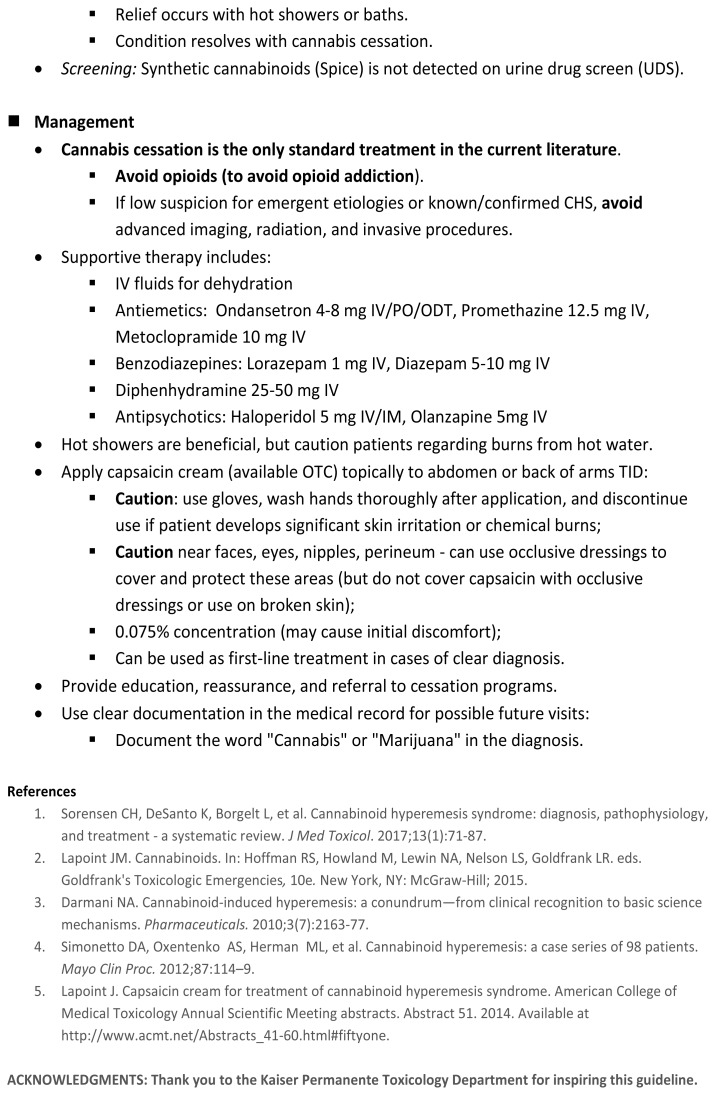
Cannabinoid hyperemesis syndrome expert consensus treatment guideline. *°C*, degrees Celsius; *CHS*, cannabinoid hyperemesis syndrome; *ED*, emergency department; *GI*, gastrointestinal; *IM*, intramuscular; *IV*, intravenous; *ODT*, orally disintegrating tablet; *OTC*, over the counter; *PO*, orally; *TID*, three times a day; *TRPV1*, transient receptor potential (vanilloid) cation channel 1; *UDS*, urine drug screen.
